# Heat Transfer Analysis of Nanocolloids Based on Zinc Oxide Nanoparticles Dispersed in PEG 400

**DOI:** 10.3390/nano12142344

**Published:** 2022-07-08

**Authors:** Alina Adriana Minea, Wael M. El-Maghlany, Enass Z. Massoud

**Affiliations:** 1 Materials Science and Engineering Faculty, Technical University Gheorghe Asachi, 700050 Iasi, Romania; 2 Mechanical Engineering Department, Faculty of Engineering, Alexandria University, Alexandria 21544, Egypt; elmaghlany@alexu.edu.eg; 3 Mechanical Engineering Department, Arab Academy for Science, Technology and Maritime Transport, Alexandria University, Alexandria 21544, Egypt; enass.massoud@gmail.com

**Keywords:** zinc oxide, PEG, numerical, heat transfer, convection

## Abstract

Cooling and heating are extremely important in many industrial applications, while the thermal performance of these processes generally depends on many factors, such as fluid flow rate, inlet temperature, and many more. Hence, tremendous efforts are dedicated to the investigation of several parameters to reach an efficient cooling or heating process. The interest in adding nanoparticles in regular heat transfer fluids delivered new fluids to the market, the nanofluids. In this paper, a new nanoparticle-enhanced fluid based on polyethylene glycol with ZnO nanoparticles is considered and its hydrothermal performance is investigated for HVAC applications. The thermophysical properties of PEG 400—ZnO and their variation with temperature at different nanoparticle loading are previously determined on experimental bases and here implemented in a numerical application. The numerical results are completed at Reynolds number from 200 to 2000, while the nanoparticle concentration varies from 0.5 to 5%. Results are discussed in terms of Nusselt number, friction factor, and dimensionless pressure drop ratio at different temperatures and ZnO loading in the PEG 400 base fluid. Additionally, the evaluation performance criteria (EC) are calculated and discussed. Concluding, the newly developed fluid enhances the heat transfer up to 16% with a 13% pressure drop penalty, while the performance evaluation criteria are enhanced. Plus, several correlations are developed for both Nusselt number and friction factor as a function of relevant operating conditions.

## 1. Introduction

Cooling and heating processes play a prominent role in industrial applications, starting with heavy industry and going to microprocessors and other applications. The thermal performance of cooling and heating processes generally depends on many factors, such as fluid flow rate, inlet temperature, and many more. Therefore, a lot of researchers have investigated the effects of such parameters to reach an efficient cooling or heating process. On the other hand, nanomaterials attracted the attention of many scholars due to their extraordinary properties. Henceforth, numerous research efforts have been accomplished to investigate the behavior of nanomaterials in various fields of mechanical engineering, while the attention was also focused on developing new heat transfer fluids, namely nanofluids. For many years, nanofluids research was focused mainly on alumina and carbon-based nanofluids, while in the last years, research has been extended to other nanoparticles (i.e., SiO_2_, CuO, TiO_2_, ZnO). Research on ZnO-based nanofluids is in its pioneering phase and a few studies are reviewed here, considering different ZnO nanoparticle applications. ZnO nanoparticles attracted noteworthy research consideration owing to their capacity for advancing thermophysical properties qualities, as well as the tribological properties, and are capable of a very good dispersion feature [1,2,3,4,5,6,7]. Plus, ZnO nanoparticles are cheaper than other nanoparticles and have the advantages of easy accessibility, biocompatibility, and biodegradability, which makes them appropriate for large-scale applications [8]. Nanofluids with water and ZnO were considered by Rahmati [1] to experimentally investigate the effects of packing density on the thermal performance of a mechanical draft wet cooling tower. Different concentrations, up to 0.1%, have been manufactured to study the impact of nanoparticles on the thermal performance of the wet cooling tower. The conclusion indicates that irrespective of using water or nanofluid, the thermal performance remains dependent mainly on the number of packing layers, however, it slightly depends on the nanoparticle concentration (i.e., around 11% positive effect on cooling efficiency for 0.1% ZnO addition). ZnO nanoparticles are also considered an additive for diesel oil and the research performed by Mousavi et al. [2] revealed that the friction factor can be improved by nanoparticles (NPs) in a concentration of 0.4 wt%, and the pumping power was augmented by nanoparticle integration in the base fluid. Dinesh et al. [9] examined both the thermophysical and the tribological characteristics of engine oil with ZnO and the conclusion is that the nanoparticles addition is able to improve the operational facets of the engine oil. Esfe et al. [10] performed experiments on engine oil using MWCNT and ZnO and concluded that the nano-additives in the engine oil can decrease the damage to motor sections in a cold start of the vehicle. Wen et al. [8] investigated the thermal and flow characteristics of two volumetric concentrations (i.e., 0.75 and 1.5%) of ZnO dispersed in water in two multiport mini-channels and noticed an enhancement in heat transfer rate at higher Re number (i.e., Re = 1430) of up to 48%. Moreover, the influence of symmetric and mixed chevron angles on the thermal performance of ZnO nanofluid in a plate heat exchanger was studied by Kumar et al. [11]. The authors demonstrate that the ideal improvement in thermal performance befell at 1% concentration of ZnO nanoparticles, in the detriment of larger concentrations. Ali et al. [12] examined the cooling process of a car radiator with ZnO nanofluids, and the best results are attained at 46% for a 0.2% volume concentration of zinc oxide. McCants et al. [13] used ZnO nanofluid for their experiments on a heated plate and observed a cooling improvement of 1.6% volume concentration of zinc oxide due to low density and viscosity of ZnO nanofluid. Another application of ZnO nanofluids was studied by Islam et al. [14], who used the suspension to cool PEM fuel cells and demonstrated a 29% improvement in the cooling capacity of the 0.5 vol% nanofluid. Sharifpur et al. [15], Li et al. [16], Hozien et al. [17] and a few other researchers demonstrate the heat transfer benefits of water, EG and mixtures between water and EG-based nanofluids with ZnO nanoparticles. Asadi and Pourfattah [18] studied ZnO in combination with engine oil for heat transfer capabilities in terms of thermophysical properties and conclude that nanofluids based on ZnO-engine oil can be beneficial in a limited range of temperatures.

Another area to focus is the refrigeration process, and nanofluids have been studied lately as an option to classical refrigerants (i.e., mainly due to their less environmentally friendly profile) since the key characteristic of nanofluids is their capability to improve heat transfer without shifting the Newtonian performance of the fluid while nanoparticles are added [19,20]. By definition, refrigerants have the capability to absorb heat at low temperatures and pressures and yield to higher temperatures and pressures [19]. In this idea, a useful review of nanofluids application, including refrigeration, was completed by Okonkwo et al. [21], where it can be seen that a number of nanofluids, mostly based on water, EG, or different oils can successfully replace classical refrigerants in several applications (see, for example, the experimental work of Ahmed and Elsaid [22], as well as [23,24,25]). Nevertheless, no ZnO nanofluid has been studied by now in refrigerant systems.

Despite the demonstrated benefits of both ZnO nanoparticles and ZnO-based nanofluids, no studies were performed using polyethylene glycol (PEG) as the host fluid. PEG can be successfully used as heat transfer fluid for numerous applications and the research is still in the development phase [26,27,28]. Basically, PEG is to be used especially in applications to replace EG for toxicity reasons and its main application is in refrigeration systems and HVAC. Plus, if compared with similar refrigerants, PEG has the disadvantage of high viscosity, however, the advantages of increased thermal conductivity and specific heat [26] can surpass the drawbacks.

To the authors’ best knowledge, research on PEG plus ZnO nano-enhanced fluids is not available, and this study can be considered a step forward to state-of-the-art knowledge. Therefore, in this study, a numerical approach is considered, having as the starting point several experimental studies on PEG 400—ZnO fluid thermophysical properties. This approach is as accurate as possible, considering that the new fluid is implemented as a single-phase one with experimentally determined characteristics (see [29,30]), as is highly recommended in the current literature (see Nanoround benchmark study [31]).

In this study, laminar forced convection flow of PEG 400 and PEG 400—ZnO nanofluid in a horizontal tube under constant heat flux is considered and numerically investigated based on real thermophysical data previously experimentally determined. Thus, the single-phase homogeneous model can be successfully employed (see Minea et al. [31] for a full explanation on selecting the most appropriate numerical approach in nanofluid simulation). Results are discussed in terms of Reynolds number and nanoparticle concentration effect on both the heat transfer efficiency and pressure drop. Entropy generation investigation is also argued to identify the benefits and drawbacks of using PEG-based nanofluids.

## 2. Thermophysical Properties of the Nanofluids

The considered new fluid is zinc oxide nanoparticles dispersed in PEG 400 (base fluid) as a pioneer new fluid (i.e., instead of ethylene glycol) in air conditioning and refrigeration applications. The new fluid is tested experimentally (see [29,30] for a full description in terms of thermophysical properties) with thermophysical properties as given below.

(1) $$\frac{\mu_{\mathrm{nf}}}{\mu_{f}}=1+5.065\phi +518.84\phi^{2}$$

The isobaric heat capacity experimental values for nanofluids were correlated as:

(2) $$\frac{c_{p\;\mathrm{nf}}}{c_{p\;f}}=1.052-0.068\frac{T}{T_{0}}+8.989\phi -381.755\phi^{2}+4668.889\phi^{3}$$

The thermal conductivity of the nanofluid is given by:

(3) $$\frac{k_{\mathrm{nf}}}{k\;f}=-1606.6\;\phi^{2}+24.733\;\phi +1.0213$$

In Equations (1)–(3), µ is the viscosity, ϕ is the volume fraction, and k is the thermal conductivity. The subscripts are as follows: f refers to the PEG base fluid, and nf refers to the nanofluid.

Equations (1)–(3) are experimentally correlated based on the outcomes already available in the literature [29,30]. As a short description, the chemicals are acquired from Merck (Darmstadt, Germany) and Propylene glycol PEG 400 (Kollisolv^®^ PEG E 400, CAS number 25322-68-3) and ZnO (CAS number 1314-13-2). The suspensions are rigorously calculated, and thermophysical properties are determined at both heating and cooling, in the temperature range 293.15–333.15 K. The thermal conductivity experiment employs the transient hot-wire technique using a KD 2 Pro Thermal Properties Analyzer (Decagon Devices, Pullman, WA, USA). The KD2 equipment are calibrated, and the accuracy of the experiment has been within ±1%. The uncertainties are calculated, and the relative error is assessed as 1.48%. The rheological properties of all fluids are obtained with a rheometer Physica MCR 501 (Anton Paar, Graz, Austria), while their expanded uncertainty is declared as 5%. The isobaric heat capacity (cp) is measured with the help of a Mettler Toledo differential scanning calorimeter, DSC 1, (Mettler Toledo, Columbus, OH, USA). All the experimental results were carefully discussed and compared with state-of-the-art, proving their validity (see [29,30] for the full explanations and comments). As an outline of the experimental outcomes [29,30], it was found that PEG 400 thermal conductivity remains constant if the temperature rises, while the addition of ZnO nanoparticles increases by 11% the thermal conductivity at ambient temperature and keeps constant the heating behavior of suspensions. In regard to the viscosity, its upsurge is sensible, being up to 13.27% for the highest concentrated suspension, while every nanofluid viscosity decreases by 10% at heating. On the other hand, the isobaric-specific heat augmentation is between 1.5 and 5.4%, depending on ZnO loading.

## 3. Mathematical Modeling

**Figure 1:**
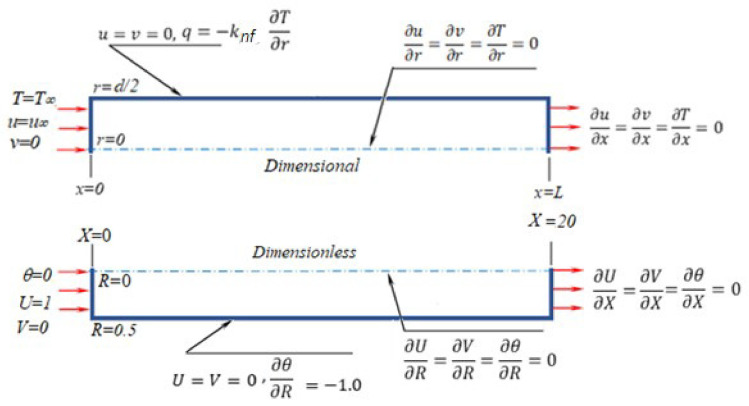
Schematic diagram in dimensional and dimensionless forms.

The problem description with boundary conditions is given in Figure 1 in both dimensional and dimensionless forms. The tube length is 20 times higher than the tube diameter (i.e., L = 20 d) to ensure fully developed flow. The flow is considered laminar with constant uniform heat flux on the outside tube surface. The problem is two-dimensional in both radial and axial directions without swirl flow in steady state flow conditions with body forces and viscous dissipation neglected. 

The governing equations in two-dimensional form are given as:

Continuity equation

(4) $$\frac{1}{r}\frac{\partial \left(\mathrm{rv}\right)}{\partial r}+\frac{\partial u}{\partial x}=0\;$$

Momentum equation

- r-direction:
  (5) $$\rho_{\mathrm{nf}}\left(v\frac{\partial v}{\partial r}+u\frac{\partial v}{\partial x}\right)=-\frac{\partial p}{\partial r}+{\;\mu }_{\mathrm{nf}}\left[\frac{1}{r}\frac{\partial }{\partial r}\left(r\frac{\partial v}{\partial r}\right)-\frac{v}{r^{2}}+\frac{\partial^{2}v}{\partial x^{2}}\right]\;$$
- x-direction:
  (6) $$\rho_{\mathrm{nf}}\left(v\frac{\partial u}{\partial r}+u\frac{\partial u}{\partial x}\right)=-\frac{\partial p}{\partial x}+\mu_{\mathrm{nf}}\left[\frac{1}{r}\frac{\partial }{\partial r}\left(r\frac{\partial u}{\partial r}\right)+\frac{\partial^{2}u}{\partial x^{2}}\right]\;$$

Energy equation:

(7) $$\left(v\frac{\partial T}{\partial r}+u\frac{\partial T}{\partial x}\right)=\alpha_{\mathrm{nf}}\left(\frac{1}{r}\frac{\partial }{\partial r}\left(r\frac{\partial T}{\partial r}\right)+\frac{\partial^{2}T}{\partial x^{2}}\right)$$

The following dimensionless formulas for generality are given as

(8) R= rd,X= xd,U=u u∞,V=v u∞,P=p ρnfu∞2,θ=T−T∞qknfd,Pr=νfαf=μf cPfkf, Re=u∞dνf

The dimensional governing equations via the formulas in Equation (8) will be

(9) $$\frac{\partial U}{\partial X}+\frac{1}{R}\frac{\partial \left(\mathrm{RV}\right)}{\partial R}=0$$

(10) U∂V∂X+V∂V∂R=−∂P∂R+μnfρfμfρnf1Re1R∂∂RR∂V∂R−VR2+∂2V∂X2

(11) U∂U∂X+V∂U∂R=−∂P∂X+μnfρfμfρnf1Re1R∂∂RR∂U∂R+∂2U∂X2

(12) $$V\frac{\partial \theta }{\partial R}+U\frac{\partial \theta }{\partial X}=\frac{\alpha_{\mathrm{nf}}}{\alpha_{f}}\frac{1}{\mathrm{RePr}}\left(\frac{\partial^{2}\theta }{\partial R^{2}}+\frac{\partial^{2}\theta }{\partial X^{2}}\right)$$

If one considers Figure 1, the boundary conditions are given as:

▪ At x = 0, T = T_∞_, u = u_∞_ and v = 0
▪ At x = L, $\frac{\partial u}{\partial x}=\frac{\partial v}{\partial x}=\frac{\partial T}{\partial x}=0$
▪ At r = 0, $\frac{\partial u}{\partial r}=\frac{\partial v}{\partial r}=\frac{\partial T}{\partial r}=0$
▪ At r = d/2, u = v =0 and $q\;=-k_{nf}\frac{\partial T}{\partial r}$

while the boundary conditions in dimensionless form (see Figure 1) are:

▪ At X = 0, θ = 0, U = 1 and V = 0
▪ At X = L/d = 20, $\frac{\partial U}{\partial X}=\frac{\partial V}{\partial X}=\frac{\partial \theta }{\partial X}=0$
▪ At R = 0, $\frac{\partial U}{\partial R}=\frac{\partial V}{\partial R}=\frac{\partial \theta }{\partial R}=0$
▪ At R = 0.5, U = V =0 and $\frac{\partial \theta }{\partial R}=-1.0$

The heat transfer from the tube outside surface heat flux to the inner fluid by convection is equal to the conduction heat transfer through the first fluid layer in contact with the tube interior surface as follows

(13) $$q=h\left(T_{w}-T_{b}\right)=-k_{\mathrm{nf}}{\left(\frac{\partial T}{\partial r}\right)}_{r=\frac{d}{2}}$$

The local Nusselt number is given by:

(14) $${\mathrm{Nu}}^{*}=\frac{\mathrm{hd}}{k_{f}}$$

Accordingly,

(15) $${\mathrm{Nu}}^{*}=\frac{q}{(T_{w}-T_{b)}}\frac{d}{k_{f}}=\frac{k_{\mathrm{nf}}}{k_{f}}\frac{1}{\theta_{w}-\theta_{b}}$$

where

(16) $${\left(\theta_{b}\right)}_{X}={\left(\frac{\int_{0}^{0.5}U\theta \mathrm{dR}}{\int_{0}^{0.5}\mathrm{UdR}}\right)}_{0\leq X\leq 20}$$

The local Nusselt number in Equation (15) with the definition of the bulk temperature in Equation (13) is integrated along the pipe length to attain the average Nusselt number as:

(17) $$\mathrm{Nu}=\overset{20}{\underset{0}{\int }}\frac{k_{\mathrm{nf}}}{k_{f}}\frac{1}{\theta_{w}-\theta_{b}}\mathrm{dX}$$

The average friction factor is calculated as:

(18) f=∫020μnfμf8Re∂U∂RdX

All the symbols significance is detailed in the nomenclature at the end of this research manuscript.

## 4. Numerical Solution Procedure

The governing equations in dimensionless form with their boundary conditions are solved numerically via the finite volume technique. An in-house FORTRAN-based code is constructed to solve the problem with FVM that has been developed by Patankar [32]. The governing equations have been converted from nonlinear forms to linear algebraic equations over the control volumes and have been solved iteratively. The numerical solution must go through three sequence steps prior to the results output: Namely grid independence, solution convergence, and, finally, code validation. The convergence of the iterations is determined by the inconsistency in velocities in radial and axial directions in addition to the Nusselt number and friction factor through one hundred iterations to be less than 0. 01% from their initial values. Three thousand iterations are more appropriate for establishing convergence. In order to confirm the independence between the grid and the numerical results, the governing equations are solved with three different mesh grids (100 × 85), (90 × 75), (80 × 65), (70 × 55), and (60 × 45). Table 1 gives the average Nusselt number and friction factor at different operating conditions and grid sizing. A suitable grid size to solution convergence with less time consumption at 3000 iterations for convergent steadiness is 80 × 65.

**Figure 2:**
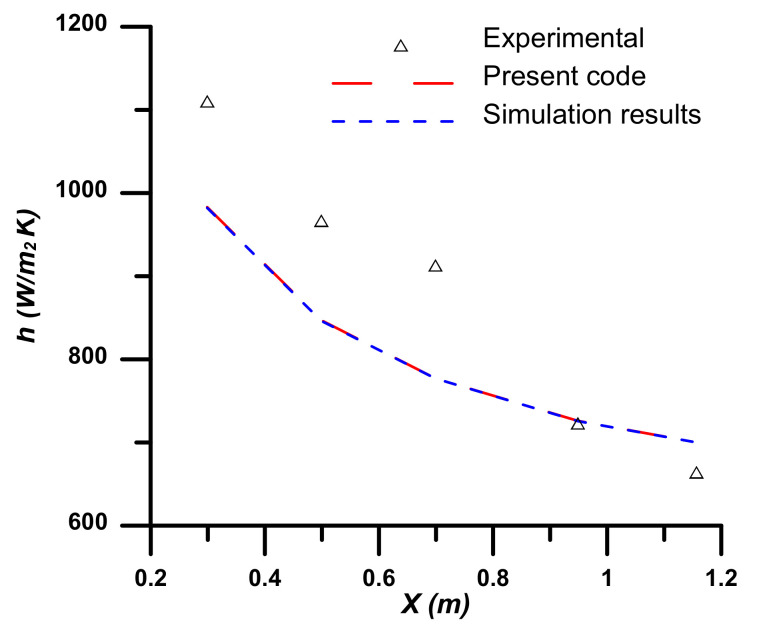
Validation of the present code local heat transfer coefficient with experimental [33] and simulation work [34].

The developed in-house numerical code is validated with both experimental [33] and numerical [34] results (see Figure 2). 

The local heat transfer coefficient along the axial direction (*x*) at the tube surface is compared. The working fluid is a nanofluid with water as base fluid and Al_2_O_3_ nanoparticles in laminar flow fully developed. The nanoparticle concentration is 4%, and the tube is subjected to uniform constant heat flux. Good agreement with the numerical solution [34] is found and has acceptable agreement with the experimental results [33]. The large deviation between the numerical code and the experimental results is due to the less accurate estimation of the nanofluid thermal conductivity.

## 5. Results and Discussion

Results are further discussed in terms of Nusselt number, friction factor, and dimensionless pressure drop ratio at different temperatures and ZnO loading in the PEG 400 base fluid. Additionally, the evaluation performance criteria (EC) are calculated and discussed. The Reynolds number varies from 200 to 2000, while the nanoparticles concentration varies from 0.5 to 5%. The thermophysical properties are experimentally determined in the temperature range from 303.15 to 333.15 K, as affirmed previously.

**Figure 3:**
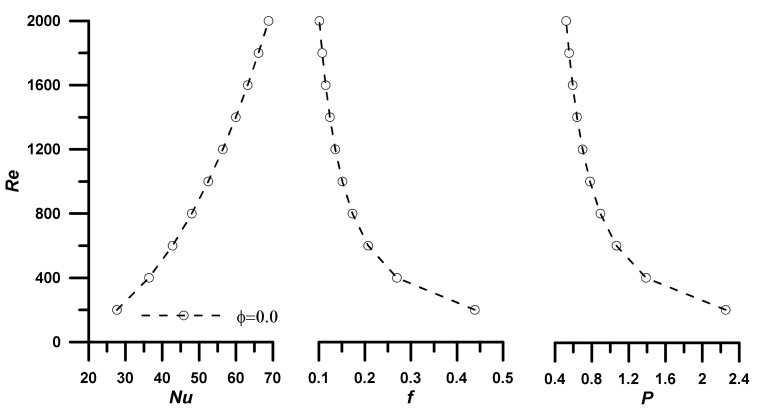
Nusselt number, friction factor, and dimensionless pressure drop at T = 303.15 K and ϕ = 0.0.

**Figure 4:**
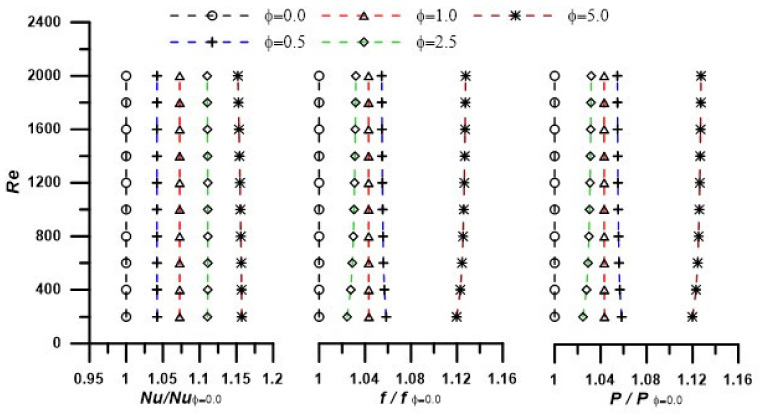
Nusselt number ratio, friction factor ratio, and dimensionless pressure drop ratio at T = 303.15 K for investigated nanofluids.

The results in the forms of Nusselt number (Nu), the friction coefficient (f), the dimensionless pressure drop (P), and the evaluation performance criteria (EC) are to be introduced as a function of Reynolds number (Re) as the main parameter in forced convection heat transfer and fluid flow in the considered tube configuration (see Figure 1). The output results are introduced at different operating temperatures due to the strong thermal dependence of the thermophysical of the nanofluid. The results are first discussed at ϕ = 0.0 (base fluid) and, after that, in terms of the performance ratio having this case as a reference (i.e., the case of the base fluid: ϕ = 0.0). Figure 3 depicts the effect of Re number on Nu, f, and P, at T = 303.15 K. As expected, the Nusselt number increases with the increase in the Reynolds number, while the friction factor and dimensionless pressure drop decrease in agreement with the classic heat transfer problem for flow inside a tube in laminar forced convection. With the addition of nanoparticles, the fluid thermal conductivity is enhanced ($\frac{k_{\mathrm{nf}}}{k_{f}}>1$) by 10.5% (see Table 2), and so the Nusselt number is augmented (Equation (17)) according to the thermal conductivity enhancement ratio ($\frac{k_{\mathrm{nf}}}{k_{f}}$, which is mainly dependent on the nanoparticles concentration (ϕ)). The peak enhancement in the heat transfer was noticed as 16% at ZnO concentration of ϕ = 5% (see results depicted in Figure 4). On the other hand, the penalty of the viscosity increases due to the addition of nanoparticles ($\frac{\mu_{\mathrm{nf}}}{\mu_{f}}>1$) up to 15.3% is translated as an increase in both friction factor and pressure drop up to 13% at ZnO concentration of 5%. Hence, these authors think that calculating of an evaluation criterion (EC) is extremely relevant, as will be discussed later. The same scenarios are repeated as the temperature increases (up to 333.15 K). However, the variation in the thermal conductivity with temperature is not remarkable, and a minor enhancement in the heat transfer is observed. On the other hand, the viscosity is decreased as the temperature increases, and the friction factor and pressure drop penalty are also decreased with temperature increase.

The addition of nanoparticles enhanced the nanofluid thermal conductivity and, consequently, the heat transfer rate, however, the viscosity was also increased, and associated pressure drop was established, which represents a non-desirable penalty. The evaluation performance criteria (EC) represent the ratio between the heat transfer enhancement due to the utilization of nanoparticles to the associated pressure drop penalty and is defined as:

(19) $$\mathrm{EC}=\frac{{\mathrm{Nu}}_{\mathrm{nf}}}{{\mathrm{Nu}}_{f}}/{\left(\frac{f_{\mathrm{nf}}}{f_{f}}\right)}^{\frac{1}{3}}$$

**Figure 5:**
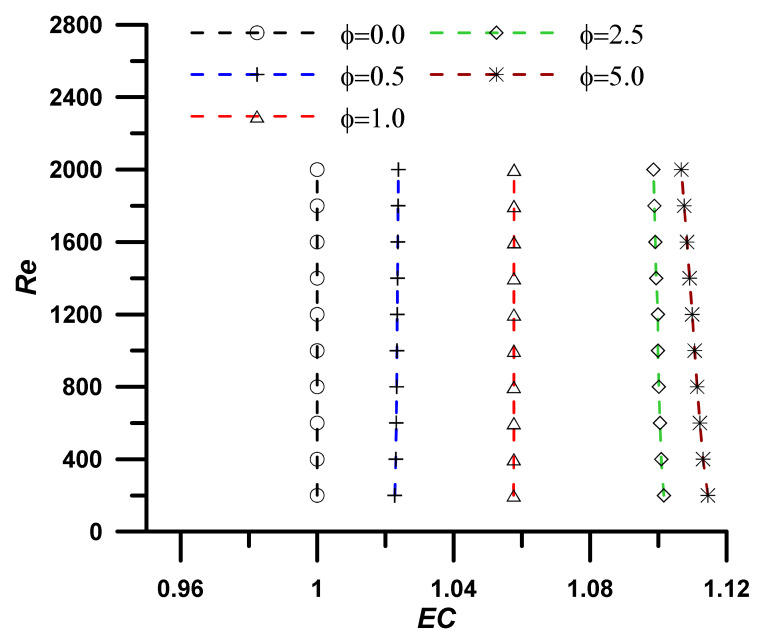
Evaluation performance criteria at T = 303.15 K for investigated nanoparticle concentrations.

**Figure 6:**
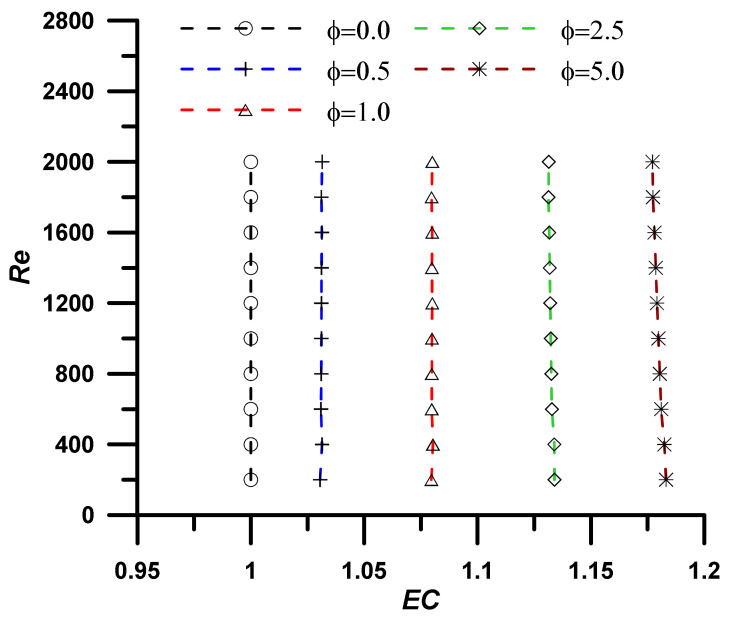
Evaluation performance criteria at T = 313.15 K for investigated nanoparticle concentrations.

**Figure 7:**
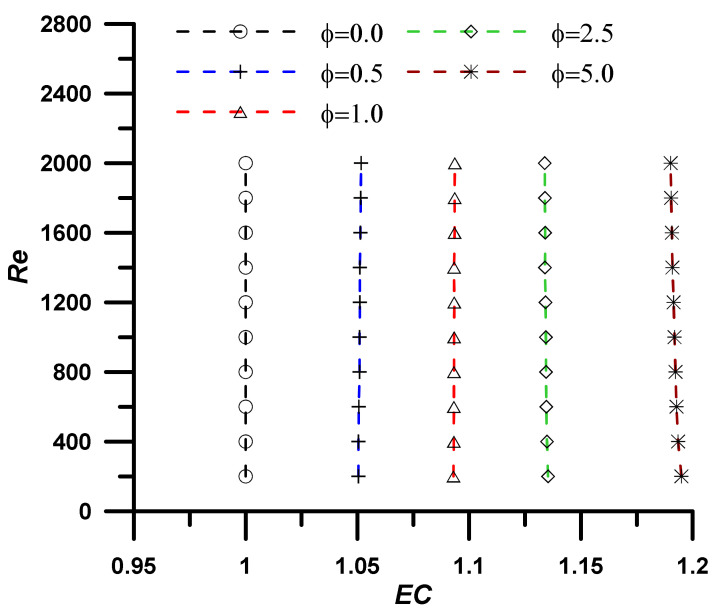
Evaluation performance criteria at T = 323.15 K for investigated nanoparticle concentrations.

**Figure 8:**
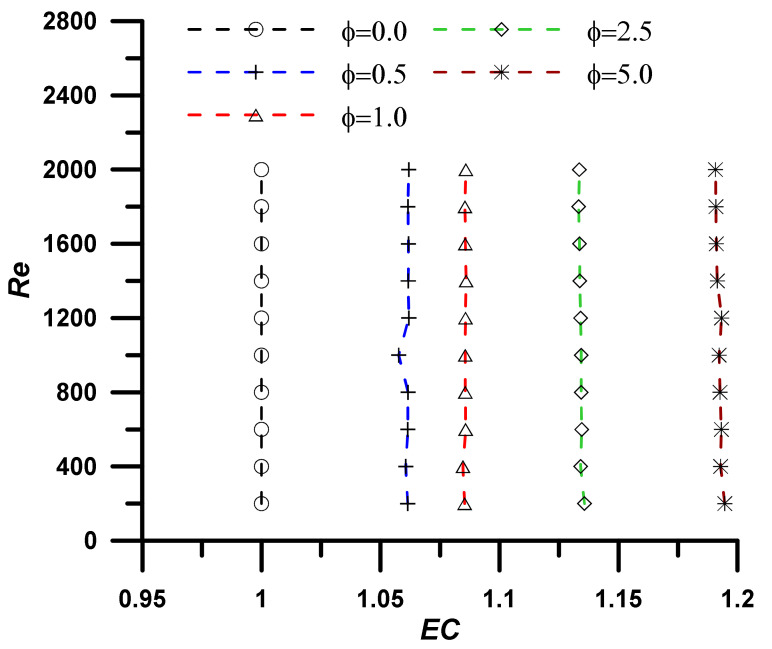
Evaluation performance criteria at T = 333.15 K for investigated nanoparticle concentrations.

An EC value greater than unity refers to high heat transfer enhancement with respect to the pressure drop penalty, and the utilization of the nanofluid instead of the base fluid is a successful choice. The results, as outlined in Figure 5, Figure 6, Figure 7 and Figure 8, show that the EC is greater than unity for all nanoparticles concentration showing amplified values as the concentration of the nanoparticles increase. It should also be noted the EC increase at high operating temperature due to the fluid viscosity decrease (i.e., following the normal decrease of viscosity while temperature upsurge).

If we consider a certain operating temperature and nanofluid volume concentration, the increase in Reynolds number is attained due to the velocity increase. Hence, as the velocity increases, the inner forces increase without any additional effect on the viscous flow. This phenomenon appears due to the fluid viscosity forces. Therefore, the boundary layer near the tube wall is diminished as a large temperature gradient appears at the first layers of the fluid near the heat source (∂T/∂r). Consequently, the heat transfer by convection will increase, resulting in an enhancement in the Nusselt number. On the other hand, a minor effect of viscous force due to viscosity appears with respect to interior force due to velocity at specified thermophysical properties of the fluid. This leads to a decrease in friction factor, as was noticed in this study.

As the nanoparticle concentration increases, the thermophysical properties of the fluid will be affected. More exactly, an increase in both fluid viscosity and thermal conductivity is noticed (see Refs. [29,30] for the full description of thermophysical properties variation). Hence, due to the fact that the Reynolds is evaluated at fluid properties, the effect of Re increase is minor, and its influence on heat transfer will mainly depend on the enhancement of thermal conductivity, also considering the pressure drop penalty that appears due to viscosity upsurge.

**Figure 9:**
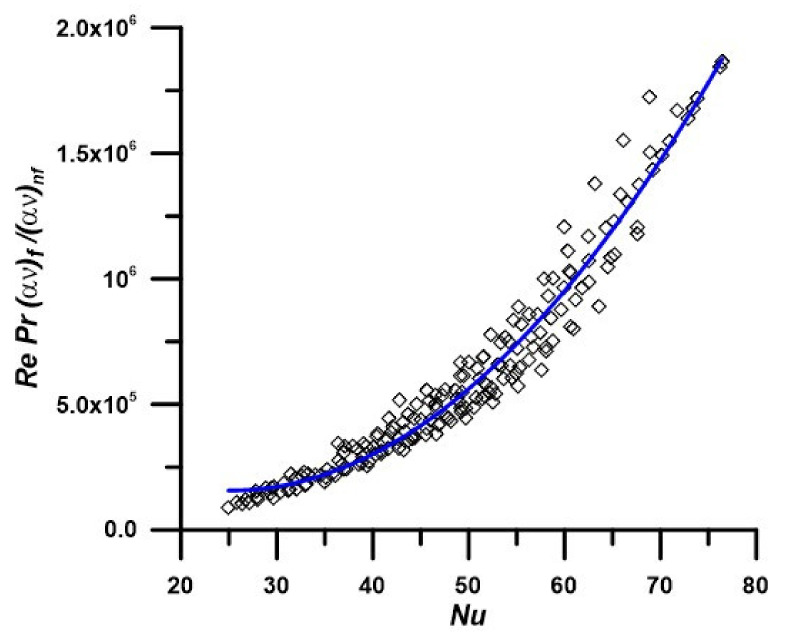
Average Nusselt number at different nanofluid properties depending on temperature and nanoparticle concentration.

As mentioned above, many parameters affect the hydrothermal performance of these new nano-enhanced fluids, for example, Reynolds number (Re), nanoparticles concentration (ϕ), temperature, nanoparticles size, and thermophysical properties, as well. In this regard, these authors attempt to combine all parameter effects in one correlation for both the Nusselt number and friction factor coefficient. The nanoparticle size influence was not investigated here since just one powder type (CAS number 1314-13-2) was considered, as was explained before. In this particular case, the study validity is restricted to ZnO nanoparticle size of about 90–100 nm. Figure 9 shows the Nusselt number variation versus all parameters described as a main thermal term, defined as *δ* and is correlated as:

(20) $$\mathrm{Nu}=22+5.8\times 10^{-5}\;\delta -1.76\times 10^{-11\;}\delta^{2}\;$$

where δ=Re Pr ανfανnf expresses the main thermal term, as used for the correlation depicted as Equation (19).

**Figure 10:**
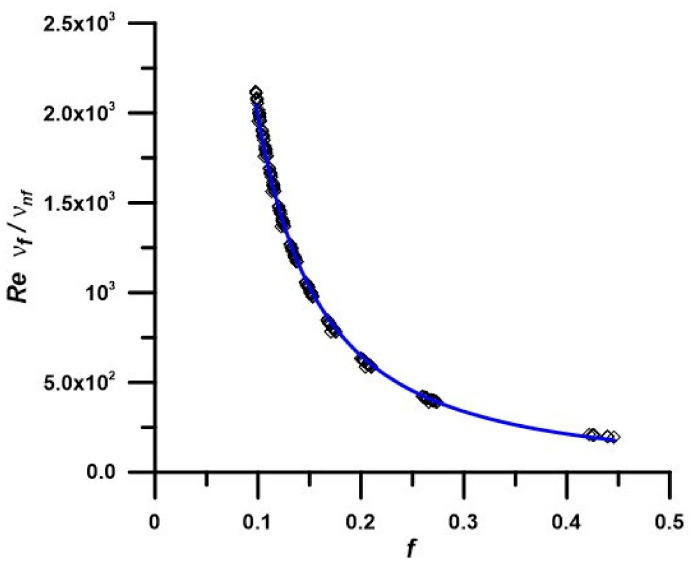
Average friction factor at different nanofluid properties depending on temperature and nanoparticle concentration.

Plus, following the same approach, Figure 10 shows the results in terms of the friction coefficient variation versus the parameters that are involved in the hydrodynamic term, Ω and is correlated as:

(21) $$51.24={\Omega \;f}^{1.583}$$

where Ω=Re ν fνPnf expresses the main hydrodynamic term, as used for the correlation depicted as Equation (20).

In Figure 9 and Figure 10, the points represent the numerical results for all nanofluids, while the line is the correlated equation. The correlations are valid for PEG 400—ZnO nano-enhanced fluids with mass concentrations up to 5%. The maximum deviations between the numerical results and correlated values are 10.1 and 4% for the Nusselt number and average friction factor, respectively (see also results plotted in Figure 9 and Figure 10).

## 6. Conclusions

In this numerical study with direct application to HVAC systems, several suspensions based on PEG 400 enhanced with ZnO nanoparticles were investigated in terms of their flow behavior in tube geometry. The nanoparticle size influence was not investigated here, and the authors cannot firmly say if the size has a major influence, thus the study validity is restricted to ZnO nanoparticle size of about 90–100 nm. The nano-enhanced fluids were implemented in an in-house code based on Fortran that was validated carefully, and all the fluids properties were previously experimentally determined. All these conditions can guarantee the stiffness of the results, creating the premises for a valid numerical approach.

Summarizing, the main conclusions of this research are:

- The Nusselt number increases with nanoparticle addition, mainly due to the thermal conductivity enhancement. The peak enhancement in the heat transfer was noticed as 16% for ZnO concentration of ϕ = 5%, combined with a penalty in viscosity of up to 15.3%.
- The proposed criteria for the evaluation of the nanofluid performance at heat transfer (i.e., EC) showed that PEG 400 can be successfully replaced by PEG 400—ZnO suspensions.
- EC increase at high operating temperature mainly due to the fluid viscosity decrease.
- New correlations for the Nusselt number and friction factor are proposed. These correlations, based on both experimental and numerical data, can be very useful for predicting the behavior of these new fluids both in heat transfer and in service (i.e., friction factor).

As a general conclusion, ZnO nanoparticles, even if less studied, are a good opportunity for heat transfer enhancement, combined with low viscosity increase, which makes them good candidates for real-life applications.

### Prospectives

This research results encourage the use of new nanofluid based on PEG and ZnO in HVAC systems, as well as other PEG-based fluids, nevertheless, further experimental work is needed to demonstrate if these new fluids can be readily used in real life applications. Plus, an interesting study may also consider different nanoparticle sizes and check their influence on both flow and heat transfer performance. Another important point to be further attained is the environmental profile of these PEG–ZnO fluids, especially if accidental damage to the HVAC system may occur. Nevertheless, experimental checks are mandatory to be able to draw a solid conclusion on different real-life cases.

## Figures and Tables

**Table 1:** The grid independence at ϕ = 0.5 and different Reynolds number.

	Grid Size									
	(60 × 45)		(70 × 55)		(80 × 65)		(90 × 75)		(100 × 85)	
Re	Nu	f	Nu	f	Nu	f	Nu	f	Nu	f
400	28.2101	0.261	29.213	0.269	29.308	0.2715	29.308	0.2715	29.308	0.2715
1000	41.61	0.143	42.121	0.15	42.661	0.1521	42.661	0.1521	42.661	0.1521
1800	53.781	0.091	54.281	0.098	54.329	0.1079	54.329	0.1079	54.329	0.1079

**Table 2:** Nanoparticle concentration and temperature variation on both thermal conductivity and viscosity.

	T = 303.15 K		T = 313.15 K		T = 323.15 K		T = 333.15 K	
ϕ	$\frac{\mu_{\mathrm{nf}}}{\mu_{f}}$	$\frac{k_{\mathrm{nf}}}{k_{f}}$	$\frac{\mu_{\mathrm{nf}}}{\mu_{f}}$	$\frac{k_{\mathrm{nf}}}{k_{f}}$	$\frac{\mu_{\mathrm{nf}}}{\mu_{f}}$	$\frac{k_{\mathrm{nf}}}{k_{f}}$	$\frac{\mu_{\mathrm{nf}}}{\mu_{f}}$	$\frac{k_{\mathrm{nf}}}{k_{f}}$
0	1	1	1	1	1	1	1	1
0.5	1.042	1.047	1.043	1.042	1.032	1.069	1.045	1.068
1.0	1.042	1.073	1.043	1.084	1.032	1.101	1.045	1.094
2.5	1.056	1.094	1.043	1.105	1.065	1.116	1.045	1.105
5.0	1.153	1.105	1.152	1.121	1.161	1.138	1.182	1.141

## Data Availability

Not applicable.

## Abbreviations

Nomenclature	
c_p_	Specific heat, J/kg K.
d	Pipe inside diameter, m.
f	friction factor.
h	Average heat transfer coefficient, W/m^2^ K.
k	Fluid thermal conductivity, W/m K.
L	Pipe length, m.
Nu	Average Nusselt number.
Nu*	Local Nusselt number.
p	Pressure, N/m^2^.
P	Dimensionless pressure.
Pr	Prandtl number.
q	Flux, W/m^2^.
r	Radial coordinate, m.
R	Radial dimensionless coordinate.
Re	Reynolds number.
T	Local temperature, K.
T_b_	Bulk temperature, K.
T_∞_	Inlet fluid temperature, K.
Tw	Wall temperature, K.
u	Velocity components in r direction.
v	Velocity components in x direction.
U	Dimensionless velocity component in R direction.
V	Dimensionless velocity component in X direction.
x	Longitudinal dimension coordinate, m.
X	Longitudinal dimensionless coordinate.
Greek symbols	
α	Thermal diffusivity, m^2^/s.
δ	Thermal term
θ	Dimensionless temperature.
μ	Dynamic viscosity, kg/m s.
ν	Kinematics viscosity, m^2^/s.
ρ	Density, kg/m^3^.
ϕ	Volume concentration.
Ω	Hydrodynamic term
Subscripts	
f	Base fluid
nf	Nanofluid
Abbreviations	
EC	Evaluation performance criteria
HVAC	Heating, ventilation, and air conditioning
PEG	Polyethylene glycol
